# In Situ Viscoelasticity Behavior of Cellulose–Chitin Composite Hydrogels during Ultrasound Irradiation

**DOI:** 10.3390/gels7030081

**Published:** 2021-06-30

**Authors:** Harshani Iresha, Takaomi Kobayashi

**Affiliations:** 1 Department of Energy and Environment Science, Nagaoka University of Technology, 1603-1 Kamitomioka, Nagaoka, Niigata 940-2188, Japan; s165075@stn.nagaokaut.ac.jp; 2 Department of Science of Technology Innovation, Nagaoka University of Technology, 1603-1 Kamitomioka, Nagaoka, Niigata 940-2188, Japan

**Keywords:** Ultrasound, cellulose–chitin composite hydrogel, hydrogen bonds, in situ viscoelasticity measurement, sono-response

## Abstract

Composite hydrogels with different cellulose and chitin loading were prepared, and their in-situ viscoelastic properties were estimated under cyclic exposure of 43 kHz and 30 W ultrasound (US) using a sono-deviced rheometer. US transmitted into the hydrogel caused it to soften within about 10 sec, thus causing a decline in the storage modulus (G′) and loss modulus (G″). However, when the US was stopped, the G′ and G″ returned to their initial values. Here, G′ dropped gradually in response to the US irradiation, especially in the first cycle. After the second and third cycles, the decline was much quicker, within a few seconds. When the chitin component in the hydrogel was increased, the drop was significant. FTIR analysis of the hydrogels suggested that the peaks of -OH stretching and amide I vibration near 1655 cm^−1^ shifted towards lower wave numbers after the third cycle, meaning that the US influenced the hydrogen bonding interaction of the chitin amide group. This repetitive effect contributed to the breakage of hydrogen bonds and increased the interactions of the acetylamine group in chitin and in the -OH groups. Eventually, the matrix turned into a more stabilized hydrogel.

## 1. Introduction

Cellulose and chitin are gaining attention as biocompatible polymer materials in medicine. They show superior properties which are favorable for such applications, especially in the form of hydrogels. By their nature, cellulose and chitin hydrogels have higher water retention [1,2] in a three-dimensional porous structure [3,4] formed by the cellulose or chitin network [5,6]. Moreover, the biocompatibility [7] and cytocompatibility [8] of such polysaccharide hydrogels are advantageous as tissue engineering scaffolds [8] and drug carriers [9,10]. More recently, cellulose [11] and chitin [12] hydrogel drug carriers were used as regenerative medicine hydrogels, which exhibited controllable drug release under US stimulation. Here, mimosa release from a cellulose hydrogel and gallic acid release from a chitin hydrogel were triggered by the US-promoted breakage of hydrogen bonds between the drug and the polysaccharide matrix. This meant that the US well stimulated the hydrogen-bonding networks of both hydrogels.

Recently, cellulose hydrogels were tested for US-triggered viscoelastic behavior by using an in situ sono-deviced rheometer [13]. During the analysis for evaluating viscoelastic properties, it was noted that US irradiation caused the cellulose hydrogel matrix to soften. After stopping the US, the hydrogels returned to their original viscoelastic conditions. This softening of hydrogels during US exposure will be helpful in applications like US drug delivery [14]. In addition, similar effects on the viscosity properties of US-combined aqueous polymeric systems were reported based on ex situ measurements [15,16,17]. However, an in situ analysis of the viscoelastic behavior of the cellulose hydrogels revealed direct US effects on hydrogen-bonding networks in the polymeric matrix [13]. Compared to cellulose hydrogels alone, these two polysaccharides have the possibility of forming extended, strong inter- intra- molecular hydrogen bonds in a composite cellulose–chitin polymer framework [18,19,20,21]. Even though materials developed from cellulose and chitin are composed on many inorganic and natural materials [22,23,24,25], materials from cellulose and chitin composite have a limited research history. Thus, in the present work, cellulose-chitin composite hydrogels (CCCHs) were fabricated and the US effect on their viscoelastic properties was described, based on an in situ analysis using a sono-deviced rheometer. 

## 2. Results and Discussion

### 2.1. Viscoelastic Behavior of Cellulose–Chitin Composite Hydrogel

**Figure 1:**
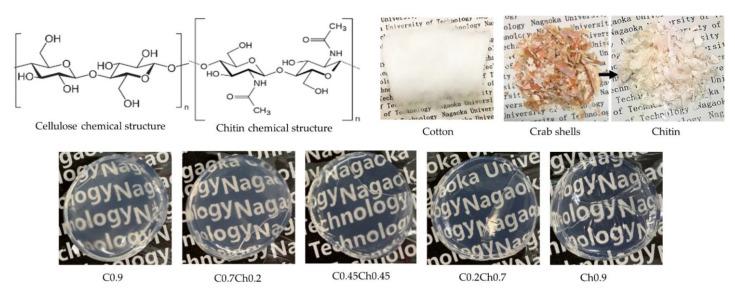
The chemical structures of cellulose and chitin, pictures of cotton, crab shells, extracted chitin, cellulose hydrogel (C0.9), chitin hydrogel (Ch0.9), and cellulose–chitin hydrogel (C0.7Ch0.2, C0.45Ch0.45, and C0.2Ch0.7).

The CCCHs were successfully fabricated by the phase inversion of cellulose–chitin solutions (CCSs), as seen in Figure 1. Here, each hydrogel was prepared from a LiCl/DMAc solution with the composition shown in Table 1. Based on cellulose-to-chitin ratios, differences in some physical properties were noted, as given in Table 2. The viscosity of the CCSs in LiCl/DMAc increased as the chitin component increased. Because of the presence of many hydrogen bonding sites, namely, methyl hydroxyl, and the acetyl amide, carbonyl and hydroxyl groups, chitin is more able than cellulose of forming strong hydrogen bonds [20,26]. Therefore, when the chitin content increased in the CCSs, it tended to create more hydrogen bonds with the cellulose as well. Thus, the solution became more and more viscous even though the total polysaccharide concentration was maintained at a constant 0.9 wt%.

In the cases of CCCHs, their appearances were transparent, as seen in the pictures, and the transparency increased when the chitin concentration was increased. The densities of the hydrogels decreased slightly with the addition of chitin. The cellulose hydrogel showed the lowest water content of 1971%, while the chitin-containing hydrogels had higher water content: 2236, 2459, 2452, and 2378% for C0.7Ch0.2, C0.45Ch0.45, C0. 2Ch0.7, and Ch0.9, respectively. These suggested that the polymeric matrix for the cellulose hydrogel was formed as a denser matrix than the chitin-contained hydrogels.

**Figure 2:**
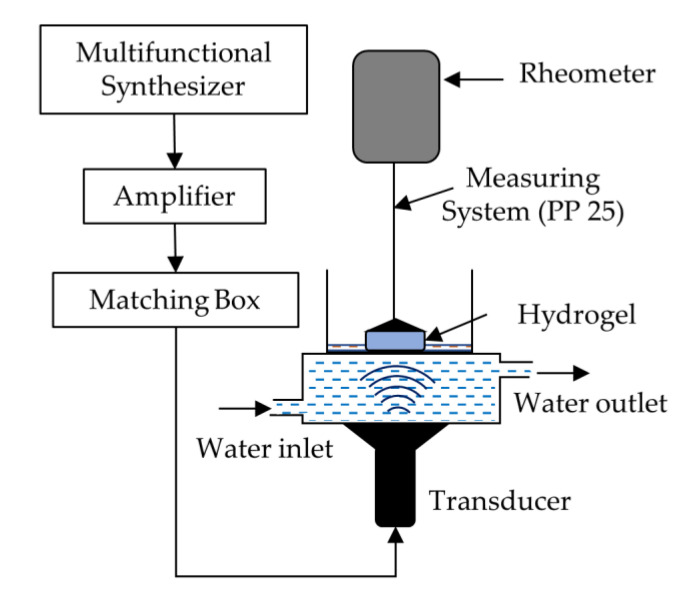
The experimental setup equipped with US transducer for in situ viscoelastic measurements.

**Figure 3:**
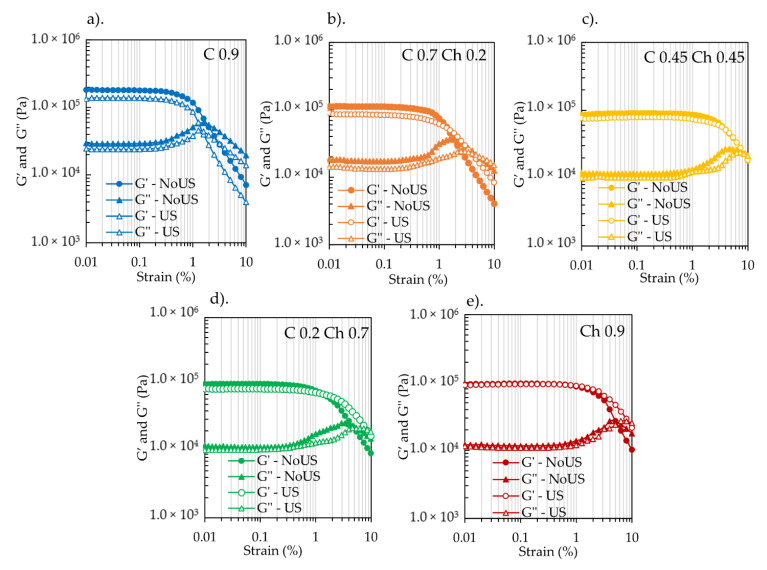
G′ and G″ of (**a**) C0.9, (**b**) C0.7Ch0.2, (**c**) C0.45Ch0.45, (**d**) C0.2Ch0.7 and (**e**) Ch0.9 before and after expose to continuous US irradiation for 1 h. The measurements were taken within 0.01–10% strain, at 1 Hz, at 25 °C. US exposure was at 43 kHz/30 W for 1 h at 25 °C.

The in situ viscoelastic properties of the hydrogels were measured during US exposure by using an experimental setup equipped with a 43 kHz US device, as shown in Figure 2 [13]. The same setup was used to perform the amplitude sweep test for the hydrogels before and after exposing them to continuous US at 30 W/43 kHz for 1 h. Figure 3 shows those viscoelastic properties at different strain % values with and without continuous US irradiation. The values of the G′ at the 0.01% strain were 1. 83 × 10^5^ Pa, 1.13 × 10^5^ Pa, 8.61 × 10^4^ Pa, 1.0 × 10^5^ Pa, and 9.4 × 10^4^ Pa, for C0.9, C0.7Ch0.2, C0.45Ch0.45, C0.2Ch0.7 and Ch0.9, respectively, without US. In the hydrogels, the highest G′ value was obtained at the lower strains %, and those values were constant up to 0.13–1.0% strain. In the case of the composite hydrogels, G′ at 0.01% strain values were decreased relative to that of C0.9. At strains higher than 1–2%, the G′ values tended to decrease because of deformations in the hydrogel structure. The G″ at lower strains stayed constant similar to the range of G′ but started to increase after strains of 1–2% up to the gel points of each hydrogel and then declined with increasing strain. Here, the gel point is the cross point of G′ and G″, i.e., G′ = G″ [1], and the strain % here was considered for the evaluations. With the increase in the chitin component, the strain % at the gel point shifted towards the higher strain end as 1.05, 1.1, 7.0, 4.0, and 5.0% for C0.9, C0.7Ch0.2, C0.45Ch0.45, C0.2Ch0.7, and Ch0.9, respectively. Briefly, it caused an increase in the elastic properties of the CCCHs. It was noted that hydrogel C0.45Ch0.45 showed the highest strain of 7.0% at the gel point, which suggested that high elasticity was maintained at higher deformation conditions. Considering the water contents given in Table 2, as explained earlier, cellulose hydrogel had a lower water content than the composite hydrogels and the chitin hydrogel. The highest water content (2459%) was seen for C0.45Ch0.45. However, the composite hydrogels had higher water retention, meaning a comparatively looser network than that of cellulose hydrogel. Among them, the C0.45Ch0.45 system seemed to have the loosest network, which meant it retained the highest amount of water while maintaining higher elastic behavior compared to the other hydrogels.

According to Figure 3, after 1 h of continuous US irradiation at 43 kHz and 30 W output power, the values of the G′ at 0.01% strain were 1.34 × 10^5^ Pa, 9.07 × 10^4^ Pa, 7.74 × 10^4^ Pa, 8.64 × 10^4^ Pa, and 9.12 × 10^4^ Pa for C0.9, C0.7Ch0.2, C0.45Ch0.45, C0.2Ch0.7, and Ch0.9 hydrogels, respectively, but all these values dropped after US exposure. After 1 h exposure, irradiation also caused the G″ values to decline. Therefore, the initial gel elasticity dropped somewhat during the long US exposure. As seen in Table 2, after 1 h exposure to US, the water content of the hydrogels also decreased, suggesting that the US released the water trapped inside the hydrogels [11].

### 2.2. Cycled US Exposure in the Viscoelasticity Change

**Figure 4:**
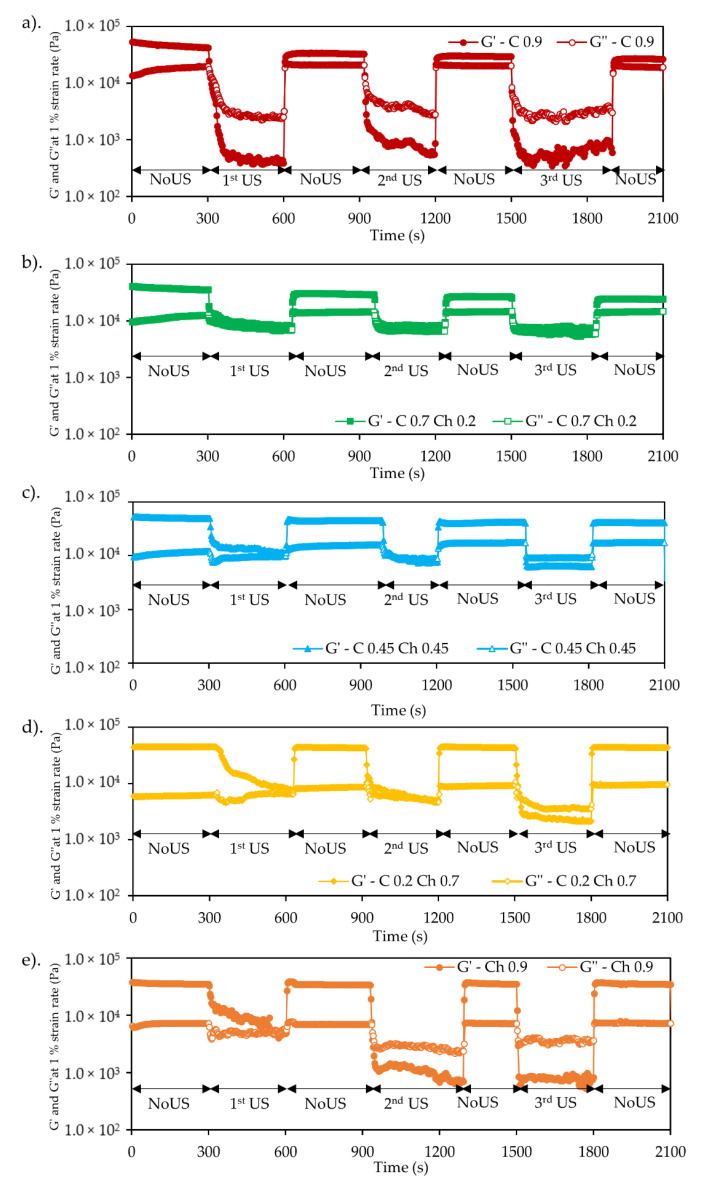
G′ and G″ vs. time plots of (**a**) C0.9, (**b**) C0.7Ch0.2, (**c**) C0.45Ch0.45, (**d**) C0.2Ch0.7 and (**e**) Ch0.9 during the cyclic US operation, i.e., in situ. G′ and G″ were measured at constant 1% strain and 1 Hz frequency. US exposure was at 30 W/43 kHz. Cyclic US and NoUS process was done at 5 min intervals.

Figure 4 shows the G′ and G″ behavior of the hydrogels under cyclic in situ US irradiation at 30 W/43 kHz. Here, the irradiation was cycled with and without US at 5 min intervals, and G′ and G″ values were monitored against the time. The G′ and G″ variations were illustrated in closed and opened symbols, respectively. For all the samples, it was clearly visible that their values started to drop once the US irradiation started. Interestingly, they fully returned to the initial values within seconds after the US stopped. This behavior happened repeatedly at three cycles of the US–NoUS steps. Here, cyclic softening and gelation behavior of the hydrogels were noted in the presence and absence of US.

The C0.9 hydrogel (pure cellulose) showed that the G′ and G″ fell sharply compared with the initial values at the NoUS steps. The cyclic behavior of the G′ decline looked similar in the first, second and third US processes. For the Ch0.9 chitin hydrogel, G′ and G″ values fell more slowly against US exposure time, especially at the first, second US cycles. It appeared that the softening effect was cycled by US exposure, meaning that US influenced the temporary breakage of the networking bonds of C0.9 and Ch0.9. The composite hydrogels showed intermediate behaviors for the G′ and G″. As given in Figure 4b–d for C0.7Ch0.2, C0.45Ch0.45, and C0.2Ch0.7, their G′ and G″ fell during the US irradiation in a similar pattern as that of Ch0.9. However, the percentage drop was not as high as either that of C0.9 or Ch0.9. It was also noted that the G′ and G″ values recovered after the US exposure stopped. Throughout the US–NoUS cycles, all values returned to their initial state, and since the values were the same, the irradiation did not break the cellulose and chitin polymer chains. Therefore, the softening effect of US might be due to changes or probably the breakage of hydrogen bonds or some physical tangles.

Interestingly, the softening behavior of the hydrogels behaved differently in the first US cycle and next to the second US cycle. The G′ dropping occurred gradually during the first cycle for C0.9 and Ch0.9, but for Ch0.9 it dropped more slowly. In the second and third cycles, the G′ values dropped more quickly than during the first, especially in Ch0.9. Considering the composite hydrogels, in the first and second cycles, the gradient of the G′ drop decreased when the chitin component increased. However, in the third US cycle, the G′ drop was almost abrupt and stayed constant over the irradiation period. The chitin hydrogel behaved similarly. This G′ and G″ behavior in the first and second cycles might be due to acetyl amine groups in the chitin. Cellulose does not have acetylamine groups or only -OH groups. Therefore, chitin-containing hydrogels may form their network with more hydrogen bonds or physical tangles, thus showing slower softening behavior during US irradiation, as explained above. Eventually, the gel matrix was rearranged into a much more stable condition under US irradiation, especially for chitin with HN-COCH3 groups.

**Figure 5:**
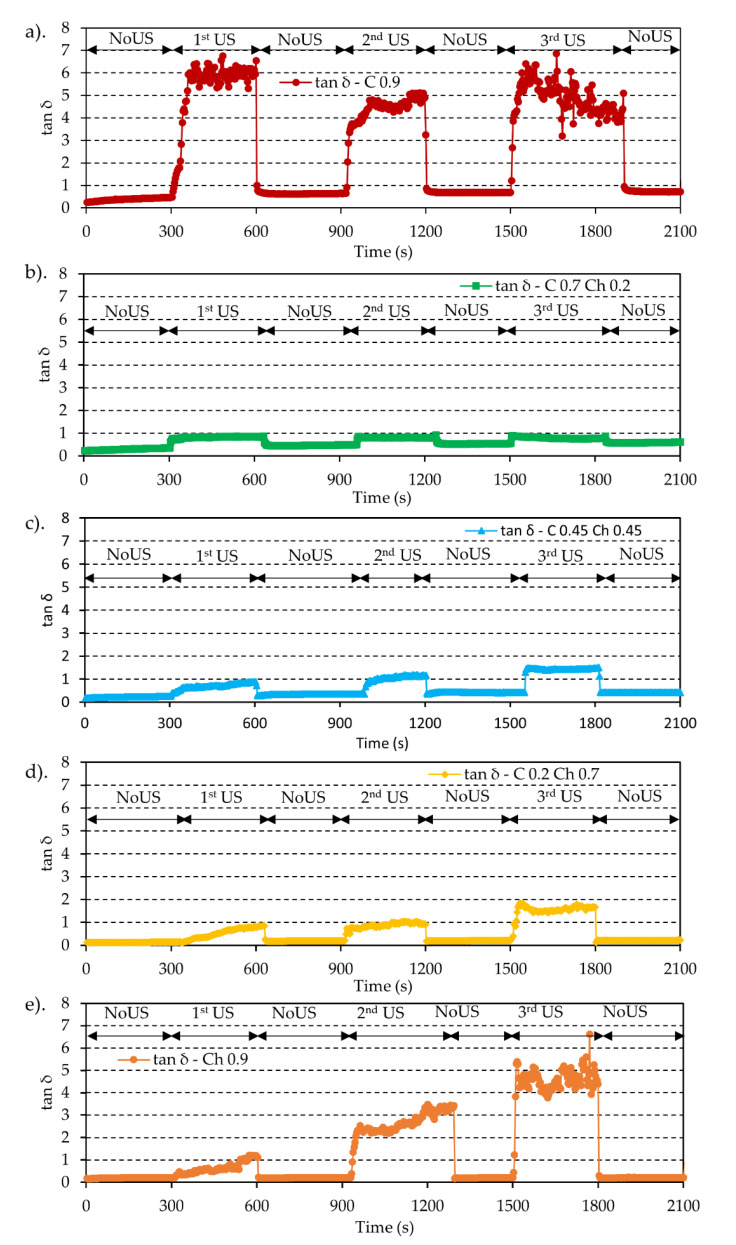
tan δ vs. time plots of (**a**) C0.9, (**b**) C0.7Ch0.2, (**c**) C0.45Ch0.45, (**d**) C0.2Ch0.7 and (**e**) Ch0.9 during the cyclic US operation.

Figure 5 shows the values of tan δ (G″/G′) variation in the cyclic US processes. When it exceeded tan δ = 1, the hydrogel became a liquid because the gel network was destroyed under the US forces. In the case of C0.9, the tan δ values were reached at tan δ ≈ 5–6, indicating a significant softening as the hydrogel turned into a liquid. In a comparison of composite hydrogels, the values of the tan δ were ≤1 for C0.7C0.2 at each US cycle. Nevertheless, there was a tendency of the tan δ value to increase with the cycles. This tendency was higher in ascending order for C0.7Ch0.2, C0.45Ch0.45 and C0.2Ch0.7. This meant that the C0.7Ch0.2 matrix kept a gel state even though the US affected the matrix bonds. In contrast, for the C0.45Ch0.45 and C0.2Ch0.7 composite hydrogels, at the first and second US cycles, the tan δ values stayed below tan δ = 1, but the values exceeded tan δ = 1 at the third. For Ch 0.9, it showed significant behavior for the same tendency. Here, the values of tan δ for Ch0.9 in the first cycle were less than those in the second or at the third, where the value tan δ was ≈5–6. Here, the Ch0.9 matrix was more sensitive to US than the composite hydrogels. However, when the US stopped, tan δ became tan δ < 1 for all the five hydrogels, meaning that the matrix returned to a gel state without permanently breaking the hydrogel network.

**Figure 6:**
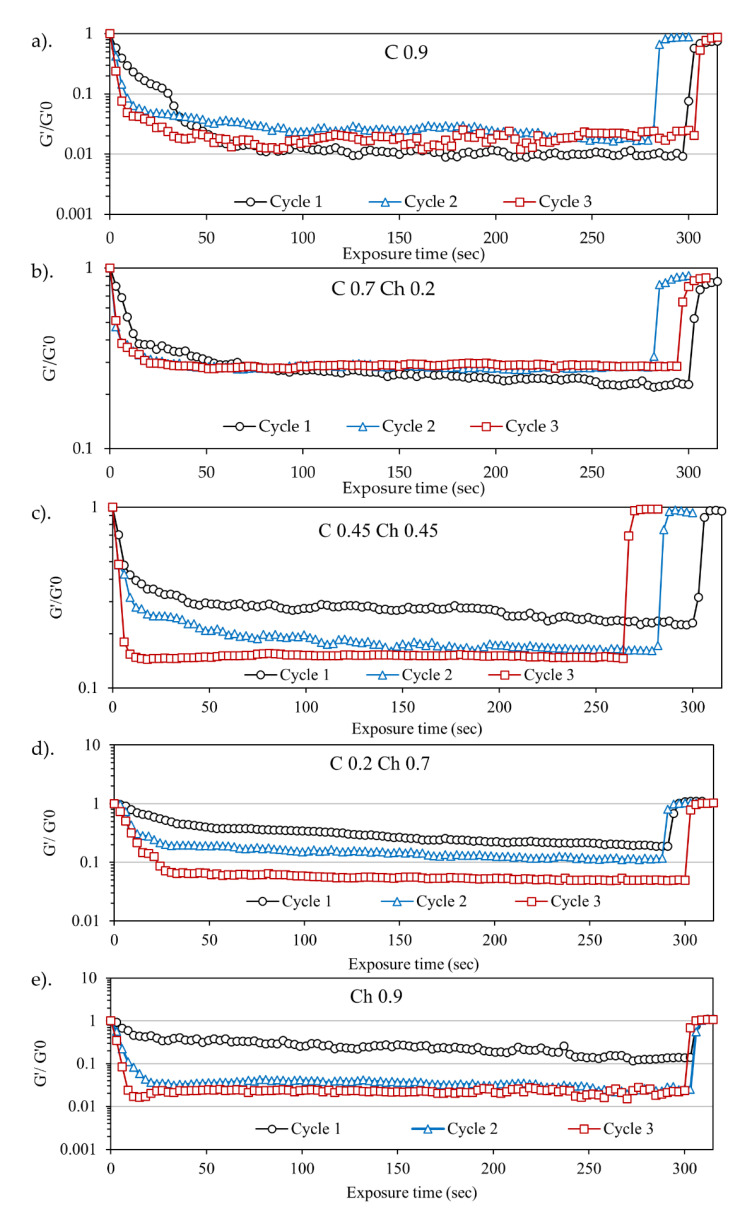
G′/G′_0_ vs. time plot during the first and second US cycles for (**a**) C0.9, (**b**) C0.7Ch0.2, (**c**) C0.45Ch0.45, (**d**) C0.2Ch0.7 and (**e**) Ch0.9. US parameters were 43 kHz and 30 W. G′_0_—initial G′ at the point of US irradiation was started. (The max. and min. points of the vertical axis scale were kept different to make the plots clearer to the reader).

To analyze the gradient changes of G′, log G′/G′0 vs. time plots were drawn as shown in Figure 6 for the first, second, and third US cycles. As per the plots, the negative slope meant the apparent softening rate of the hydrogels under US irradiation. In contrast, the positive slope referred to the bond reformation rate when the US stopped after each cycle. Here, the rates were calculated from the slope of the plots. Specifically, the rate of G′ drop or the softening rate of C0.9 for the first, second, and third irradiation cycles was 0.017, 0.058, and 0.090 s^−1^, respectively. The softening rates during the first, second, and third cycles for C0.7Ch0.2 were 0.026, 0.060, 0.060 s^−1^; for C0.45Ch0.45, 0.047, 0.056, and 0.113 s^−1^; for C0.2Ch0.7, 0.004, 0.017, and 0.028 s^−1^; and for Ch0.9, 0.004, 0.052 and 0.09 s^−1^. According to the softening rates, the values increased with successive US cycles, meaning that the matrix became progressively softer. This might be because the US released the physical tangles and hydrogen bonds of the polymer–polymer matrix stage by stage during the US–NoUS cycles. Thus, eventually, the matrix became stable. Here, when comparing different hydrogel systems, C0.45Ch0.45 exhibited the highest softening rate under US exposure. Further, the bond reformation rates when the US stopped were, for the C0.9, 0.093, 0.171, and 0.212 s^−1^ in the first, second, and third cycles, respectively. Similarly, the values for C0.7Ch0.2 were 0.089, 0.088 and 0.168 s^−1^; for C0.45Ch0.45, 0.108, 0.182 and 0.192 s^−1^; for C0.2Ch0.7, 0.163, 0.228 and 0.243 s^−1^; and for Ch0.9, 0.178, 0.202 and 0.220 s^−1^. As clearly shown, bond reformation became more efficient with each cycle. This enhanced rate of bond reformation implied that the matrix changed to be more stable with each US irradiation cycle, and when each was compared, the bond reformation rate was higher than the softening rate. Here, it was evident that hydrogel softening happened step by step due to the breakage of hydrogel linkages in the physical entanglements and hydrogen bonds. When the US stopped, re-arranging of the gel crosslinking points occurred (mostly hydrogen bond formation), so gelation occurred more quickly. This effect was more pronounced for chitin with acetylamine groups than for cellulose.

### 2.3. FTIR Analysis of Cellulose–Chitin Composite Hydrogels

**Figure 7:**
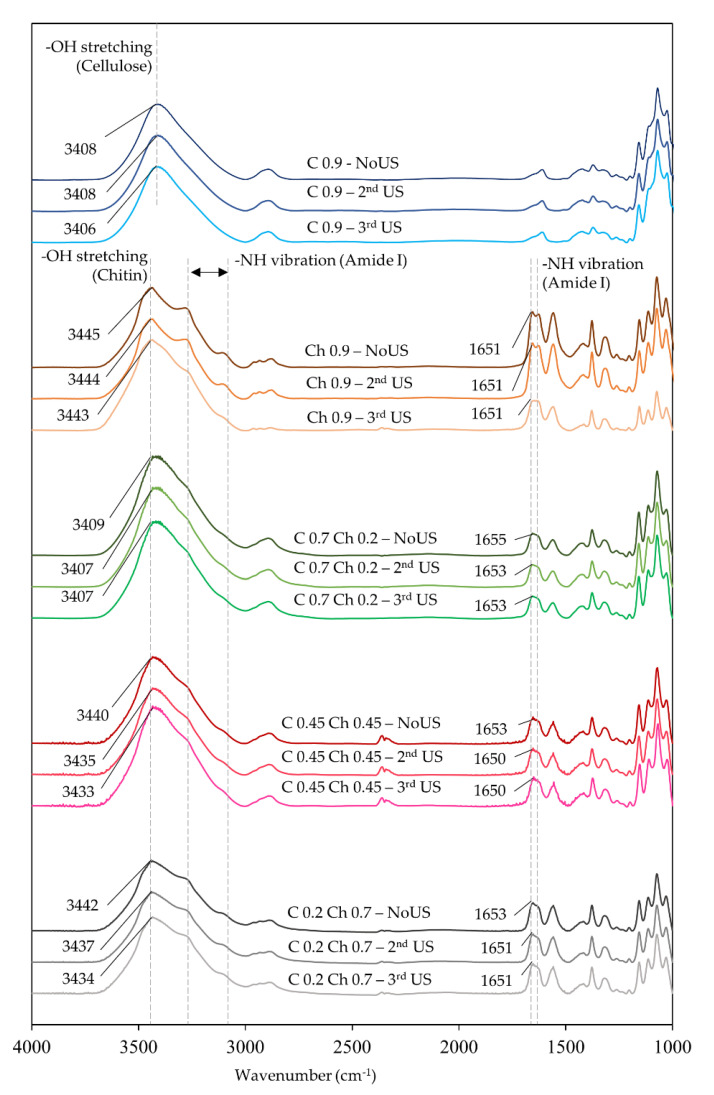
FTIR spectra of cellulose hydrogel, chitin hydrogel, and cellulose–chitin hydrogel films before US and after the third US cycle.

To confirm the US effect of the hydrogels, FTIR spectra were measured and compared before after the third US exposure (Figure 7). In the results of the -OH stretching peak, the maximum wavenumber was observed at 3408, 3409, 3440, 3442 and 3445 cm^−1^ for C0.9, C0.7Ch0.2, C0.45Ch0.45, C0.7Ch0.2 and Ch0.9, respectively. Here, the original -OH stretching peaks of cellulose and chitin at 3408 and 3445 cm^−1^ shifted towards one another in the composite hydrogels. Furthermore, the FTIR spectra of each hydrogel film when after the three US cycles showed a slight shift in the peak wavenumber towards the lower region in the -OH stretching peak and the vibration mode of Amide I at around 1650–1655 cm^−1^ in the chitin component [22,27]. This suggested that after the cycles, the wavenumber of the hydrogen bonds of the acetylamine group changed in the chitin-containing hydrogels. However, the cellulose showed no change in the first, second, and third cycles. In the chitin-containing hydrogels in the third cycle, the spectral change in the acetylamine group corresponded to the change of viscoelastic behavior. While in the first and second cycles, the drop in the G′ and the values of tan δ for chitin-containing hydrogels increased due to the release of physical tangles in the chitin segments. Then, the acetylamide groups formed other hydrogen bonds to crosslink the segment tangles. On the other hand, the cellulose and chitin composites seemed to form tight hydrogen bonds to make compacted hydrogels. However, with the increase of the chitin component, US caused changes to the tangles of chitin with acetylamine groups. Eventually, the hydrogels were arranged to be more uniform in their network.

## 3. Conclusions

Cellulose–chitin composite hydrogels were successfully fabricated using the phase inversion method. The sono-responsive nature of the hydrogels was studied using in situ viscoelasticity measuring equipment with a US device. The analytical results showed that US at 43 kHz and 30 W caused softening of the hydrogels, especially in C0.9 and higher chitin-component hydrogels. However, the gel condition immediately recovered after the irradiation stopped. When the chitin contents were increased, the softening effect was enhanced. Further, the hydrogel softening behavior was cycled during the US irradiation. However, the hydrogels with acetylamide groups delayed softening in the first US cycle. However, the second and third cycles re-arranged the segmental tangles of the hydrogels resulting in the reformation of hydrogen bonds after the US stopped.

## 4. Materials and Methods

### 4.1. Materials

Defatted cotton was a product of Kawamoto Sangyo Co., Ltd., Osaka, Japan, and was used as the cellulose source without further chemical treatment. According to our previous study, chitin was extracted from crab shells obtained from the Teradomari fish market, Nagaoka, Niigata, and purified according to our previous study [12]. For the demineralization of crab shells, hydrochloric acid (HCl) was used. Then, the deproteinization of chitin was performed from sodium hydroxide (NaOH), and the decolorization of the extracted chitin was done using ethanol (EtOH). HCl, NaOH, EtOH, Lithium Chloride (LiCl), *N, N*-Dimethylacetamide (DMAc), and Potassium Hydroxide (KOH) were purchased from Nacali Tesque, Inc. (Kyoto, Japan). Cotton was dissolved in DMAc/6 wt% LiCl solvent to obtain 1 wt% of cellulose solutions. DMAc was dried in KOH for 3 days at room temperature, and LiCl was vacuum dried for 24 h at 80 °C before the preparation of cellulose or chitin solutions.

### 4.2. Extraction of Chitin from Crab Shells

Chitin was extracted according to the former reports available [12]. The dried and crushed crab shells were stored in the freezer to maintain their original status. First, 30 g of raw crab shells were stirred with 900 mL of 1 N HCl for 24 h as the demineralization step. Then, the treated crab shells were filtered using a metal mesh and washed using distilled water till pH = 7. Next, demineralized crab shells were treated with 900 mL of 1 N NaOH for 5 h at 90 °C for deproteinization. Again, the treated crab shells were washed until neutral pH. Next, the sample underwent decolorization by stirring with an excess of EtOH at 60 °C for 5 h. Finally, the chitin was vacuum dried at room temperature for 24 h before making chitin solutions.

### 4.3. Fabrication of Cellulose–Chitin Composite Hydrogels

According to the fabrication procedure of composite hydrogels, cotton and chitin were first dissolved individually in 6 wt% LiCl/DMAc solvents of each cellulose or chitin solution with 1 wt% concentration according to our previous work [6,8]. First, 1 g of cellulose/chitin underwent three solvent exchange steps being stirred in 150 mL of distilled water, EtOH, and DMAc, for 24 h in each solvent. After the solvent exchange steps, the cellulose or chitin was vacuum dried for 24 h at 25 °C. Meanwhile, the 6 wt% LiCl/DMAc solvent was prepared by dissolving 6 g of dried LiCl in 93 mL of dried DMAc. Then, the vacuum-dried cellulose or chitin was fed into the prepared 6 wt% LiCl in DMAc solvent. This was stirred until completely dissolved to obtain a clear solution. Once the dissolution was completed for cellulose or chitin, the solutions were centrifuged at 10,000 rpm to remove undissolved cellulose or chitin and other impurities.

For the composite hydrogels, the cellulose and chitin solutions were mixed by stirring for 48 h at room temperature. As listed in Table 1, the weight ratios of cellulose (C) and chitin (Ch) were 0.7/0.2, 0.45/0.45, and 0.2/0.7 in 0.1 parts of LiCl/DMAc solutions for C0.7Ch0.2, C0.45Ch0.45 and C0.2Ch0.7 samples, respectively. Using those mixed solutions, three kinds of composite hydrogels of C and Ch were prepared as follows. First, 10 g from the cellulose–chitin mixture was poured into a 50 mm diameter petri dish and kept in a sealed container in a water vapor atmosphere at 25 °C for 24 h. During this period, the gelation process occurred, and thus the cellulose–chitin composite hydrogel was formed by phase inversion [1,21]. Then the hydrogels were washed well using 20 mL × 30 times of distilled water to remove LiCl and DMAc.

The water content of the prepared hydrogels were evaluated by the equation ((W_w_ − W_d_)/W_d_) × 100%, where the W_w_ and W_d_ are the wet and the dry weight of the hydrogels, respectively. Here, the W_w_ was measured for the hydrogels immediately after the washing was completed. Before the W_w_ measurements, the hydrogels were carefully patted by a Kim wiper to absorb the surrounding water on the hydrogel surfaces. The W_d_ was measured after vacuum drying at 80 °C for 24 h. The experiments for each sample were conducted in triplicate to assure the consistency of the results. Furthermore, the densities of the hydrogels were measured using a multifunctional balance (GX-200, A&D Company Limited, Tokyo, Japan) at 25 °C and measured three times for each sample. The viscosity of the solutions was measured at 25 °C using the rheometer Physica MCR 301 (Anton Paar, Tokyo, Japan).

FTIR analysis was performed for the vacuum-dried composite hydrogel thin films using the JASCO FTIR-4100 instrument (JASCO Corporation, Japan). The films were swollen with a 2 µl drop of distilled water and then sandwiched between two clean CaF_2_ plates (30 mm Ø × 2 mm). The opening was well sealed using a sealing tape (0.1 mm × 13 mm, SAN-EI, Osaka, Japan). The FTIR spectra were recorded for the hydrogel films before starting the irradiation: three 5 min US cycles with 5 min NoUS intervals.

### 4.4. In situ Viscoelastic Measurements Using the Sono-Devised Rheometer

A schematic diagram of the sono-devised rheometer Physica MCR 301 (Anton Paar, Tokyo, Japan) is shown in Figure 2. It was used to modify the sample platform on the bottom with a Langevin-type HEC-45242 M transducer (Honda electronics Co. Ltd., Utsunomiya, Japan), that was located vertically upwards. The signal generated via the wave factory (15 MHz WF1943B multifunction synthesizer, NF, Yokohama, Japan) was amplified by the high-speed bipolar amplifier (DC-1MHz/10GVA HAS 4032, NF, Yokohama, Japan). Then, the signal was sent through the wave homogenizer (Honda Electronics, Yokohama, Japan) and finally to the transducer equipped with a circulating water bath (86 mm × 65 mm d × h). Here, the bottom of the water bath was covered by the SUS 316 plate (0.2 mm thickness) and hinged well onto the top surface of the transducer. The hydrogel sample was put on the SUS 316 plate during the in situ viscoelasticity measurement. To ensure the wetting of the hydrogel and to ease the US transmission to the sample, 5 mL of distilled water was poured onto the sample plate. Water circulation entirely filled the water bath without any voids, and thus US transmission was not interrupted. Water bath temperature was controlled at 25 °C by using a temperature controlling water circulator. The transduced US wave was passed through the water medium and transmitted via the SUS plate and then through the sample. Deformations were not noticed on the SUS plate due to US transmittance. US was operated at a constant frequency of 43 kHz and power of 30 W. The viscoelasticity measurement for the cellulose–chitin composite hydrogels was carried out using the parallel plate 25 (PP25) measuring system, at the constant 1% strain rate and 1 Hz oscillatory frequency. Irradiation was cycled in 5 min intervals with and without exposure to US written for US and NoUS, respectively. The in situ measurements of the G′ and the G″ were recorded with time during the cycling for each hydrogel with an average 25 mm diameter and 2.5 mm thickness.

## Figures and Tables

**Table 1:** Composition of the cellulose–chitin solution (CCS) mixture used to prepare respective cellulose–chitin composite hydrogels (CCCHs).

Sample Name	Composition of CCSs Used to Fabricate CCCHs		
	1 wt% Cellulose (wt%)	1 wt% Chitin (wt%)	6 wt% LiCl/ DMAc (wt%)
C0.9	0.9	0	0.1
C0.7Ch0.2	0.7	0.2	0.1
C0.45Ch0.45	0.45	0.45	0.1
C0.2Ch0.7	0.2	0.7	0.1
Ch0.9	0	0.9	0.1

**Table 2:** Viscosity of CCSs mixtures, water contents (wet basis) of CCCHs before and after US irradiation (30 W/43 kHz, 1 h), and density of CCCHs.

Sample Name	Viscosity (25 °C, at 1% Strain) (Pa.s)	Water Content (%) (Dry Basis)		Density (g/cm^3^)
		Before US	After US	
C0.9	0.209	1971	1915	1.016
C0.7Ch0.2	0.218	2236	2171	1.014
C0.45Ch0.45	0.512	2459	2380	1.014
C0.2Ch0.7	1.14	2452	2443	1.013
Ch0.9	1.82	2378	2316	1.011
